# A novel method for the preparation of reproducible, stable, and non-infectious quality control materials for *Chlamydia trachomatis* nucleic acid detection

**DOI:** 10.1128/spectrum.00837-24

**Published:** 2024-10-07

**Authors:** Jing Li, Kuo Zhang, Yanxi Han, Rongxue Peng, Lin Li, Jinming Li, Guigao Lin

**Affiliations:** 1 National Center for Clinical Laboratories, Institute of Geriatric Medicine, Chinese Academy of Medical Sciences, Beijing Hospital/National Center of Gerontology, Beijing, China; 2 Graduate School, Chinese Academy of Medical Sciences and Peking Union Medical College, Beijing, China; 3 Beijing Engineering Research Center of Laboratory Medicine, Beijing, China; MultiCare Health System, Tacoma, Washington, USA

**Keywords:** cell lines, *Chlamydia trachomatis*, CRISPR/Cas9, nucleic acid amplification test, quality control

## Abstract

*Chlamydia trachomatis* (CT) is a significant sexually transmitted pathogen known to evoke severe complications, including infertility. Nucleic acid amplification tests (NAATs) are recommended by the World Health Organization to detect CT infection. Furthermore, the establishment of methods, performance validation, internal quality control, and external quality assessment for CT NAATs necessitate the utilization of quality control materials (QCs). QCs are specimens or solutions that are analyzed for quality control purposes in a test system. In this study, we established a novel cell line that stably integrates CT amplification target sequences for producing QCs for CT NAATs. Utilizing clustered regularly interspaced short palindromic repeats/CRISPR-associated protein 9 technology, we integrated the CT plasmid-mediated sequence (comprising the full length of the cryptic plasmid and the major outer membrane protein gene, 9,136 bp) into the *MUC4* gene of HEK293T cells. Positive clones were screened through flow cytometric sorting, single-cell culture, and PCR-based identification, followed by the establishment of stable cell lines. These cells were then processed using optimized cell preservation procedures to prepare QCs. The sequence insertion copy number was confirmed by real-time quantitative PCR. This novel CT QCs demonstrate excellent clinical applicability, non-infectiousness, quantifiability, and stability. With an integrated sequence exceeding 9 kb in length, it offers exceptional flexibility for adapting to new kit developments. Furthermore, maintaining a well-defined copy number and stable shelf life, the QCs closely aligns with the quality control requirements of CT NAATs. This study presents an innovative method for preparing QCs for CT nucleic acid detection, making a valuable contribution to improving the performance of CT NAATs.

**IMPORTANCE:**

Untreated CT infections impose significant burdens on individuals and communities, underscoring the importance of early and accurate testing via CT NAATs for disease control. QCs are instrumental in identifying testing process issues. Hence, we developed a cell line integrating CT-amplified target sequences as readily accessible non-infectious QCs. These QCs boast several advantages: the integration of over 9 kb of CT sequence allows for broad applicability, allowing flexible adaptation to the development of new kits. Confirming the CT sequence copy number provides a reliable basis for QC concentration preparation and kit detection limit evaluation. Optimized preservation protocol enhances QC stability during storage, facilitating convenient shipment to clinical laboratories at ambient temperatures. In summary, our novel CT QCs offer a powerful tool for improving CT NAAT performance and present a fresh perspective on QC preparation for detecting nucleic acids from intracellular parasitic pathogens.

## INTRODUCTION

In 2020, an estimated 128.5 million new *Chlamydia trachomatis* (CT) infections among adults aged 15–49 worldwide were reported by the World Health Organization (1). Failure to promptly diagnose and treat CT due to asymptomatic, mild, or non-specific symptoms in the early phase can lead to the continued spread of the infection (2, 3). This may result in serious secondary complications, especially female infertility, and may facilitate the transmission and acquisition of HIV and other sexually transmitted infections (2–6). Consequently, precise screening and diagnosis are crucial for timely identification and management of CT infection.

The conventional cell culture, once regarded as the standard method, is no longer suitable for the demands of rapid CT detection due to prolonged culture cycles and the need for highly skilled technicians (7, 8). Assays like enzyme immunoassay, direct fluorescent antibody test, and nucleic acid hybridization, while employed, exhibit limitations in detecting a substantial proportion of infections due to their inherent lack of sensitivity (7, 9–11). Consequently, highly sensitive and specific nucleic acid amplification tests (NAATs) are recommended as a routine diagnostic method for CT infection (12–14). However, the reliability of NAATs results may be compromised by the presence of inhibitors, cross-contamination, or improper sample handling (15, 16). Implementing stringent quality control measures, including quality control materials (QCs), is imperative to identify testing issues and defects, ensuring the accuracy of clinical test outcomes (15, 17). QCs are used to evaluate or verify performance characteristics, including measurement precision, accuracy, and analytical bias that may result from reagent or instrument changes (18).

Internationally utilized QCs for CT NAATs mainly include positive clinical samples (16, 17), cell cultures (16, 19), and recombinant plasmids containing target sequences (20). Using positive clinical samples as QCs is optimal for simulating real sample testing procedures. However, the scarcity and limited availability render them unsuitable for large-scale external quality assessment (EQA) (17). This impracticality is underscored by the participation of 1,355 laboratories in the EQA initiative for the CT DNA PCR assay in 2022, as recorded by the National Center for Clinical Laboratory of China (https://www.nccl.org.cn/showEqaPlanProDetail?id=570). While cell cultures present a viable alternative for QCs due to their mass-producibility, Chalker’s study highlighted the impact of strain type on EQA results when using CT cultured in cells (19). Additionally, challenges in cultivation and biosafety level requirements limit their availability to certain quality assessment organizations (21). Plasmids containing target sequences offer an economical and reproducible source of QCs. However, they are vulnerable to degradation and fail to mimic the nucleic acid extraction process from clinical specimens (22, 23). Consequently, none of these three QCs emerges as the optimal candidate for ensuring the quality of CT NAATs.

To fulfill the requirements of mass production, reproducibility, biosafety, and comprehensive quality control for CT QCs, our laboratory previously developed a cervical epithelial cell line (HTB-SiHa) transfected with recombinant plasmids containing partial cryptic plasmid fragments (23). The non-infectious recombinant plasmids transfect cells, proficiently preventing target gene degradation by exogenous enzymes while authentically simulating the nucleic acid extraction process of pathogens, facilitating comprehensive monitoring (23). However, recent EQA activities revealed a significant concern. Some laboratories utilizing newly developed kits reported negative test results for positive QCs. Subsequent investigation unveiled that the target sequences in these new kits were not included in our previously constructed recombinant plasmid. Additionally, the transient nature of plasmid transfection in eukaryotic systems necessitates recurrent retransfection during each preparation, introducing inconvenience to the process (24).

In recent years, the clustered regularly interspaced short palindromic repeats (CRISPR)/CRISPR-associated (Cas) proteins gene-editing technology has rapidly advanced due to its notable efficiency, simplicity, and user-friendly characteristics (25). This technology has found extensive application in precisely introducing target sequences into wild-type cell lines for QC preparation in mutant gene detection (26–31). In this study, we utilized CRISPR/Cas9 technology to induce DNA double-strand breaks (DSBs), enabling the creation of a stable CT-positive cell line via non-homologous end joining (NHEJ). Common targets in CT NAATs include the cryptic plasmid, the major outer membrane protein (*MOMP*) gene, and the ribosomal RNA (rRNA) (23). The cryptic plasmid DNA is approximately 7.5 kb in length, with about 1–10 copies per cell (32). Research indicates that assays targeting the cryptic plasmid exhibit sensitivity 10–1,000 times higher than those directed at *MOMP* gene or rRNA (33). However, a CT variant with a deletion in the plasmid has been identified in Sweden, which may lead to detection failures in kits targeting cryptic plasmids (34). As a result, some kits have incorporated detection of the *MOMP* gene fragments (33). CT strains are classified into genotypes based on the outer membrane protein gene (*ompA*), which encodes the major outer membrane protein and is typically associated with clinical presentation (35). Consequently, this cell line integrates the entire length of the CT cryptic plasmid and the complete sequence of the *MOMP* gene. Demonstrating excellent performance in homogeneity, stability, and commutability validation experiments, this non-biologically infectious cell line proves to be a valuable QC for laboratory quality evaluations. Our utilization of the CRISPR/Cas9-edited cell line presents an innovative strategy for CT nucleic acid detection QC preparation.

## RESULTS

### Generating HEK293T cell lines integrating CT sequence using CRISPR/Cas9 technology

**Fig 1:**
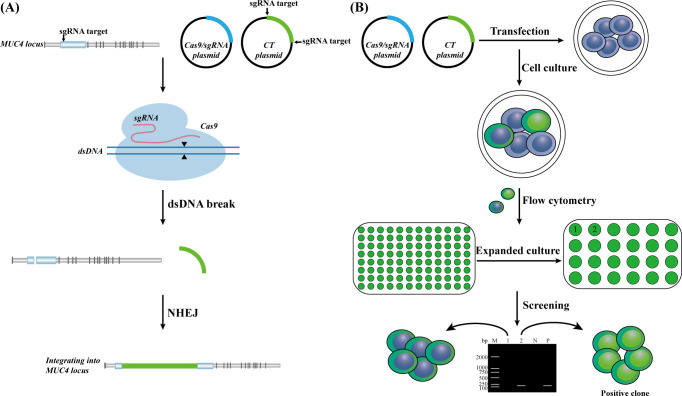
Diagram of the experimental principle and flow. (**A**) Schematic diagram of the CRISPR-Cas9 principle. The Cas9 protein binds to single guide RNA (sgRNA) to form the sgRNA-Cas9 protein complex. Guided by the sgRNA, this complex selectively binds to specific sequences, facilitating the precise cleavage of the targeted DNA and triggering the generation of double-stranded breaks. (**B**) Schematic diagram of the experimental process. Initially, HEK293T cells were co-transfected with Cas9/sgRNA plasmid and CT plasmid. Subsequently, green fluorescent protein-positive single cells were screened using flow cytometry, and positive clonal cells were ultimately identified through PCR-based agarose gel electrophoresis.

Utilizing CRISPR/Cas9 technology, we generated a HEK293T cell line integrating the entire CT cryptic plasmid sequence and the *MOMP* gene (Fig. 1). After plasmid transfection, single-cell cultures were initiated using 480 cells sorted by flow cytometry. After 2 weeks of culture, 167 cells successfully developed into monoclonal cell lines, while the remaining cells failed to survive.

**Fig 2:**
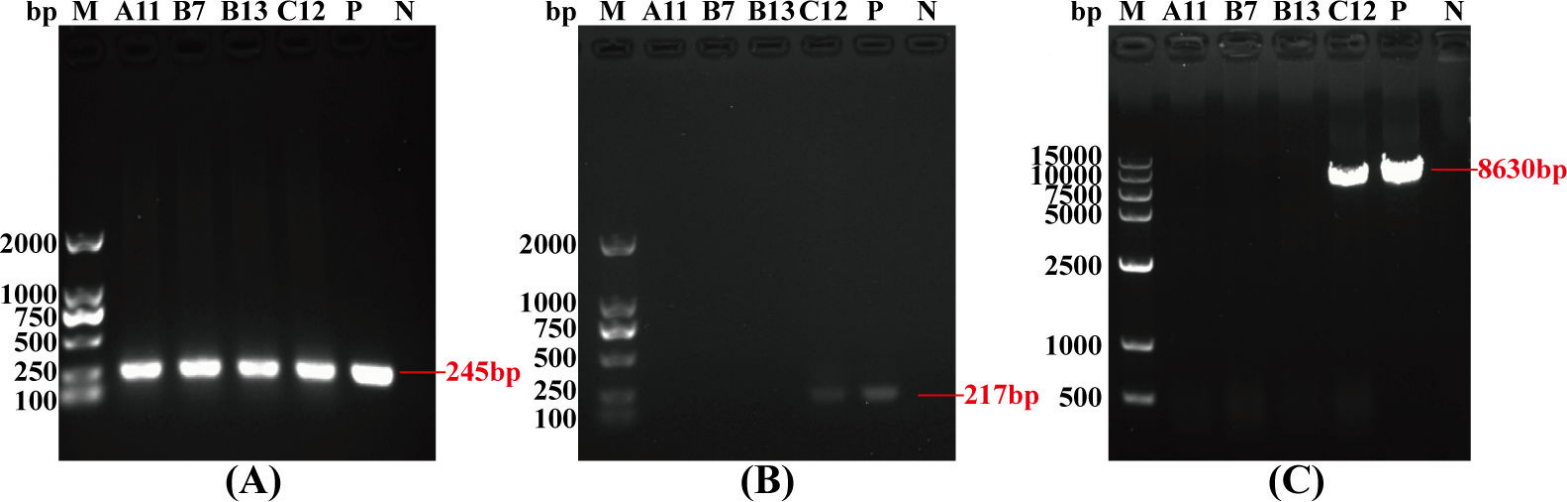
PCR-based agarose gel electrophoresis screening of positive clonal cells. (**A–C**) Screening and confirmation of four positive clonal cell lines using CT-F1/R1, CT-F2/R2, and CT-F3/R3 primers. gDNA was extracted from monoclonal cells for PCR analysis. Primer pairs CT-F1/R1, CT-F2/R2, and CT-F3/R3 amplified the head, tail, and approximate full length (245, 217, and 8,630 bp) of the CT insertion sequences, respectively. The amplification products were analyzed by electrophoresis on 1% agarose gel. The sizes of the amplicons were as expected, indicating the successful integration of the corresponding portions of the target insertion sequences into the genome. Lane M: 2,000- or 15,000-bp DNA marker; Lane N: negative control (wild-type HEK293T cells); Lane P: positive control (the residual mixed pool cells after flow cytometric sorting).

Preliminary screening using CT-F1 and CT-R1 primers successfully identified four positive clonal cell lines (A11, B7, B13, and C12) that integrated the head of the CT insertion sequence (Fig. 2A; Fig. S1). Subsequent screening with CT-F2 and CT-R2 primers confirmed that C12 was a positive clonal cell line integrating the tail of the CT insertion sequence (Fig. 2). To validate the integration of the full length of the CT insertion sequence in positive clonal cell lines, we performed PCR-based screening using primers CT-F3 and CT-R3, amplifying a fragment of 8,630 bp. The final results revealed that only C12 exhibited integration of the entire sequence of the cryptic plasmid and the *MOMP* gene (Fig. 2C). The sequenced CT sequence was 100% consistent with the designed sequence (Table S1) based on basic local alignment search tool analysis (data not shown), indicating that the entire exogenous sequence was successfully integrated into this cell line.

### Quantification of CT insertion copy number through RT-qPCR

**Fig 3:**
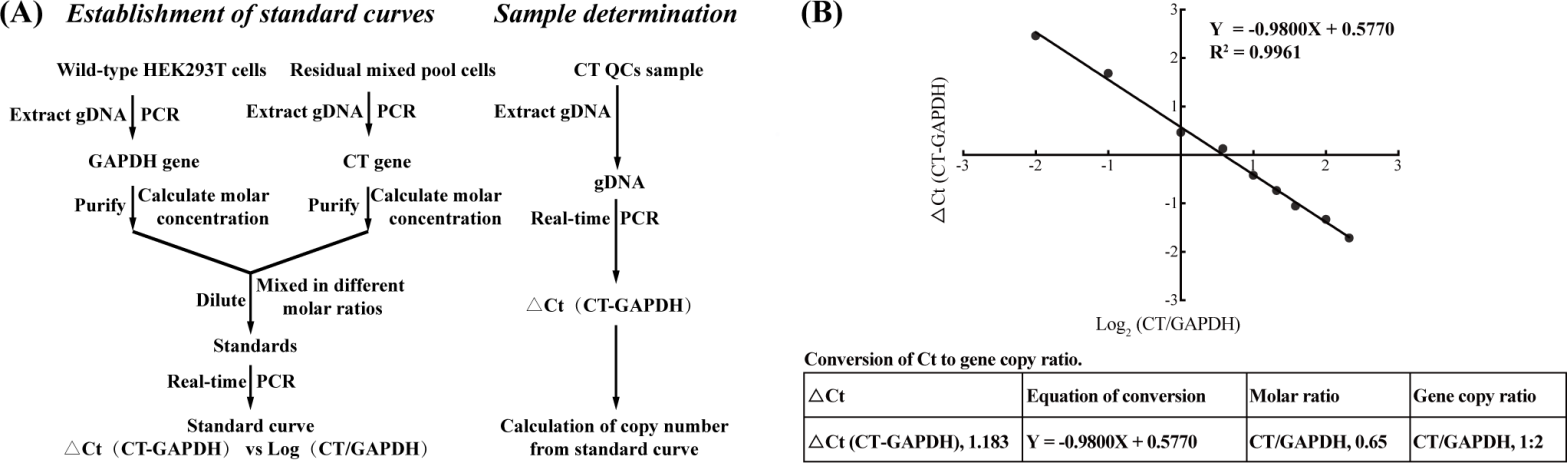
Determination of the insertion copy number. (**A**) Experimental design diagram for CT insertion copy number determination. (**B**) Standard curve and gene copy number conversion. The Ct difference vs logarithmic ratio for CT and *GAPDH*. Each data point was assessed in triplicate, and the mean value was employed to draw the graph.

We utilized real-time quantitative PCR (RT-qPCR) to determine cycle threshold (Ct) values for standards with varying molecular molar ratios of CT and *GAPDH* genes (Table S2). Subsequently, a standard curve was constructed by correlating the Ct difference with the molar logarithmic ratio (Fig. 3B). For result consistency, we determined the Ct values of the CT and *GAPDH* genes for the C12 QCs in the same batch of experiments (Table S2): 24.439 and 23.256, respectively. By substituting the ∆Ct value (1.183) into the linear equation, the calculated CT:*GAPDH* molar ratio was 0.65. HEK293T cells typically contain two copies of the *GAPDH* gene, and the CT:*GAPDH* ratio was close to 1:2 (0.65), indicating the insertion of a single copy of CT.

### Effective and sustainable preservation of cellular QC handling solutions

**Fig 4:**
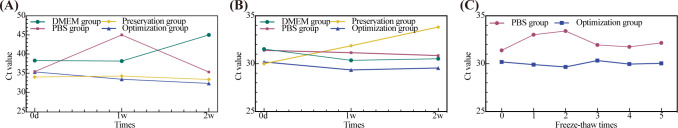
Stability assessment results of QCs prepared with different preservation solutions. (**A**) Ct values of nucleic acid extracted from four groups of QCs (10^5^ cells/m) using the extraction reagent provided by the assay kit (Hybribio, Guangzhou, China) in the 37°C stability assessment. (**B**) Ct values of nucleic acid extracted from four groups of QCs (10^5^ cells/mL) using the QIAamp DNA Mini Kit (QIAGEN) in the 37°C stability assessment. (**C**) Ct values of nucleic acid extracted from two groups of QCs (10^5^ cells/mL) using the QIAamp DNA Mini Kit (QIAGEN) in the freeze-thaw stability assessment. Ct values were determined using the CT nucleic acid detection kit (Hybribio) by Applied Biosystems 7500 Real-Time PCR Systems (Thermo Fisher Scientific, USA). Each data point was assessed once.

Analyzing the preliminary stability assessment results at 37°C (Fig. 4A), we observed significant Ct value fluctuations across three time points for QCs in the phosphate-buffered saline (PBS) and Dulbecco’s modified Eagle medium (DMEM) groups. In Fig. 4B, the Ct values of nucleic acid extracted from QCs in the preservation group gradually increased with time.

Both experiments collectively indicated that treating cells with preservation solution and ultimately preserving them with PBS offers stability and high efficiency advantages. The subsequent freeze-thaw stability experiment (Fig. 4C) further highlighted the stability advantage of the optimization group.

### Establishing homogeneous, stable, and universal CT QCs

**Fig 5:**
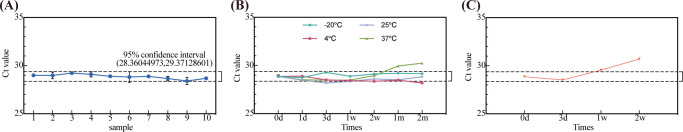
Homogeneity and stability assessment results of QCs. (**A**) Ct values for homogeneity assessment of QCs (10^5^ cells/mL). (**B**) Ct values for stability assessment of QCs (10^5^ cells/mL) at different storage temperatures. (**C**) Ct values for thermal stability assessment of QCs (10^5^ cells/mL) at 43°C. Ct values were determined using the CT nucleic acid detection kit (Daan Gene, Guangzhou, China) by Applied Biosystems 7500 Real-Time PCR Systems (Thermo Fisher Scientific, USA). Each sample underwent three repetitions under consistent conditions, and values are presented as mean ± SEM.

The homogeneity validation experiment results indicated no significant trend in Ct values (Fig. 5A). One-way analysis of variance (ANOVA) analysis (Table S3) confirmed the excellent homogeneity of our QCs.

Stability validation experiments demonstrated that our QCs maintained stability for a minimum of 2 months at −20°C, 4°C, and 25°C and for a minimum of 2 weeks at 37°C (Fig. 5B). In the thermal stability experiment, the QCs exhibited stability for at least 3 days at 43°C (Fig. 5C).

**Fig 6:**
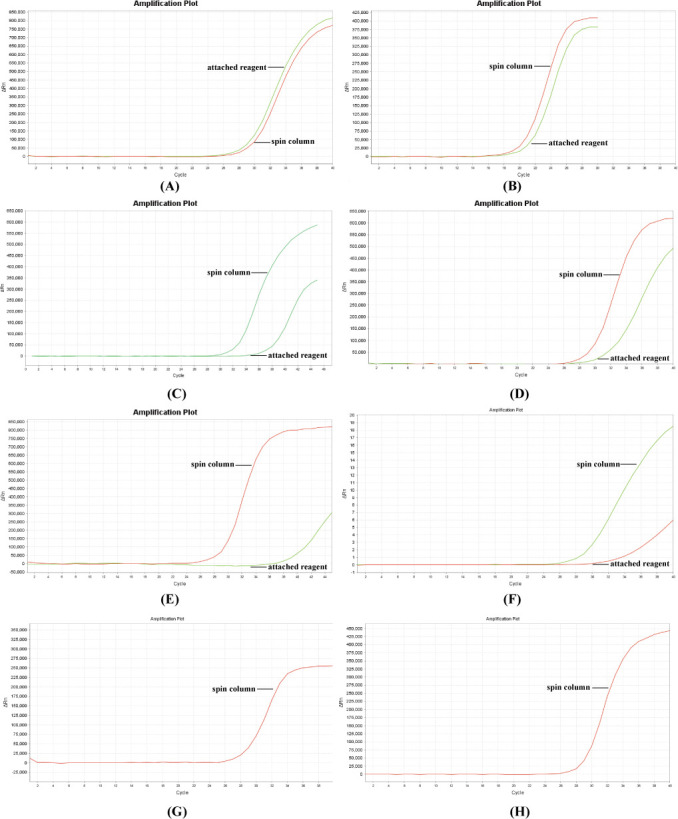
Internal commutability validation results for eight CT nucleic acid test kits. (**A**) TIANLONG (approval no. 20153400366), (**B**) Daan Gene (approval no. 20163401027), (**C**) Hybribio (approval no. 20143401937), (**D**) ACON Biotech (approval no. 20143402231), (**E**) Sansure Biotech Inc (approval no. 20153400084), (**F**) QIAGEN (approval no. 20163400008), (**G**) Daan Gene (approval no. 20213400572), (**H**) BioPerfectus Technologies (approval no. 20183400058). Each experiment simultaneously amplified QCs and positive and negative controls using Applied Biosystems 7500 Real-Time PCR Systems (Thermo Fisher Scientific, USA). The results exclusively displayed the amplification curves of QCs, omitting the positive and negative controls. Kits with approval nos. 20213400572 and 20183400058 recommended the utilization of the spin column extraction method for genomic DNA extraction. Since no accompanying nucleic acid extraction reagent was provided, the results exclusively presented the amplification curves of the spin column extraction method.

The internal commutability validation results (Fig. 6) demonstrated that all eight CT nucleic acid assay kits effectively detected the nucleic acid of our QCs, which were extracted using both lysis and spin column-based methods.

External commutability validation data (Table 1) revealed that all nine laboratories successfully identified the moderate-positive QCs and negative QCs, while six laboratories detected the weak-positive QCs. Overall, our CT QCs demonstrated excellent versatility.

**TABLE 1:** External commutability validation results^*a*^

Laboratory code	Laboratory location	Nucleic acid assay kit	Moderate-positive QCs	Weak-positive QCs	Negative QCs
A	Beijing, China	Daan Gene	+	+	−
B	Chongqing, China	Daan Gene	+	−	−
C	Guangzhou, China	Daan Gene	+	+	−
D	Guangzhou, China	Hybribio	+	−	−
E	Jinan, China	Daan Gene	+	+	−
F	Shanghai, China	Tellgen	+	+	−
G	Yantai, China	Sansure Biotech Inc	+	+	−
H	Beijing, China	Liferiver	+	+	−
I	Changsha, China	Sansure Biotech Inc	+	−	−

^*a*^ Qualitative results: positive (+) or negative (−). Moderate-positive QCs (10^5^cells/mL of CT-positive cells), weak-positive QCs (10^4^cells/mL of CT-positive cells), and negative QCs (10^5^cells/mL of HEK293T cells). The QCs are processed, tested, and analyzed by each laboratory in accordance with its standard workflow, utilizing its own instrumentation, sample handling procedure, and assay kit.

## DISCUSSION

In the realm of CRISPR/Cas9 technology for QC development, researchers have successfully utilized the CRISPR/Cas9-mediated homology directed repair (HDR) to generate QCs tailored for specific point mutations (26). However, studies indicate that CRISPR/Cas9-induced DSBs are typically repaired through NHEJ, resulting in inefficient knock-in via HDR (36). While NHEJ repair is commonly employed for targeted gene knock-out, it has been demonstrated to effectively facilitate the insertion of lengthy exogenous DNA fragments *in vitro* (36). Despite employing single guide RNA (sgRNA) targeting approximately 400 copies of repeat sequences to enhance insertion efficiency, the integration efficiency of the lengthy sequence remained low. This aligns with prior research indicating that the frequency of knock-in decreases as the size of the integrated DNA increases via NHEJ (36). The low copy number of CT sequence insertion led to an increase in both the volume of cell culture and the duration of preparation time in our QC preparation. However, refining the strategy, including optimizing the transfection protocol, utilizing multi-copy repetitive sequences for sgRNA design, and expanding the screening of single-cell clones, is anticipated to facilitate the generation of positive clonal cell lines containing multi-copy CT sequences to meet the desired requirements.

Cell lines containing amplified target sequences indeed serve as excellent NAAT QCs. However, their utility is hindered by challenges related to prolonged storage and exposure to harsh transportation conditions (37). Typically, researchers use PBS or DMEM as the main ingredient for preserving cellular QCs. However, our results suggested that QCs treated with PBS or DMEM do not provide a stability advantage in TaqMan qPCR assays during the actual quality control process. To circumvent this, researchers have innovated cell preservation solutions, often incorporating methanol as a key component (38). Methanol, acting as a dehydrating fixative, is crucial for preserving cellular morphology and structure (39). It effectively inhibits lysosomal enzyme lysis, ensuring the stability of intracellular biomolecular structures (39). Additionally, incorporating polyethylene glycol (PEG) into the preservation solution enhances stability, with its relatively low melting point reducing temperature fluctuations during QC storage (40). However, our results showed that these components might not be effectively removed during nucleic acid extraction, potentially inhibiting PCR amplification (41, 42). To mitigate potential inhibitory effects of PEG and methanol on the amplification reaction, we optimized the preservation solution treatment protocol. This optimization facilitated effective monitoring of the amplification reaction by the QCs while ensuring stable preservation.

Currently, QC concentrations are commonly quantified using commercial kits, such as Xpert CT/NG test (PCR) (Cepheid, USA), through the assessment of Ct values (16, 21). However, we utilized the homogeneity of clonal cell lines, along with the quantification of CT sequence insertions and cell numbers, to precisely determine the CT copy number in the QCs. This methodology establishes a robust foundation for QC concentration preparation and kit detection limit evaluation. Homogeneity validation experiments highlight the remarkable uniformity of our QCs, suggesting the generation of a stable QC library from a single harvest of mass-produced cells. Our QCs boast robust stability and can be conveniently shipped at ambient temperatures. This not only preserves cell integrity by avoiding potential damage associated with freeze-drying but also reduces costs by eliminating the need for dry ice transportation (19). These results collectively demonstrate that our QCs offer dependable, consistent, and enduring performance. These attributes are essential for ensuring the validity and reliability of laboratory quality control procedures. The cellular QCs, containing an exceeding 9-kb CT insertion sequence, are suitable for target detection in most commercial kits and adaptable to target sequences from newly developed kits in the future. Additionally, it offers an effective intracellular control, serving as an excellent assay matrix for kits with intracellular control targets. This feature aids in assessing sample quality and identifying potential PCR inhibitors (15). However, variations in detection efficacy were observed among kits due to differences in the composition and operating procedures of the nucleic acid extraction reagents. Laboratories should select extraction methods and commercial kits based on their specific testing requirements. Furthermore, the non-detection of some weak-positive QCs indicates the need to carefully consider factors such as nucleic acid extraction efficiency, detection sensitivity of test kits, deoxyribonuclease contamination, and technician proficiency.

While our QCs offer quantifiability, reproducibility, non-infectious nature, and stability, certain limitations persist. Firstly, our sequence construction did not incorporate rRNA targets, as commercial kits targeting 23S rRNA (e.g., Aptima CT assay, TMA; Hologic/Gen-Probe, USA) are not widely used in China. Therefore, our QCs are designed for kits detecting CT DNA and are not be suitable for those targeting RNA. This design restricts the versatility, particularly for laboratories specializing in RNA detection. Future investigations may consider introducing RNA targets to augment the versatility of our QCs across various detection kits.

Secondly, our cellular QCs with integrated CT sequence differ somewhat from actual clinical samples. For example, they do not mimic CT present outside of infected cells, and the DNA sequences of CT from truly infected cells do not integrate into genomic DNA (gDNA). Additionally, CT has a cell wall structure similar to that of Gram-negative bacteria, making it challenging to fully replicate the nucleic acid extraction process from real clinical samples (43). Furthermore, our QCs do not realistically mimic genitourinary and extragenital urine and swab samples routinely processed on instruments (19). Because it is a resuspension, monitoring the impact of sample viscosity or matrix-related issues on the extraction protocol is unattainable (19). Nevertheless, optimizing matrix dilution, such as using urine or other simulated clinical matrices, is expected to generate QCs that more closely emulate actual clinical samples. Future research in this direction will further enhance the simulation and utility of our QCs, providing laboratories more reliable QCs.

Regarding the validation of commutability, we also faced some limitations. Firstly, we did not use internationally mainstream kits such as the Abbott RealTime CT/NG Assay (PCR) (Abbott, USA) or the Cobas CT/NG Test (PCR) (Roche, USA) to validate our QCs as these kits are not commonly used in Chinese laboratories. While our study utilized only the China National Medical Products Administration (NMPA)-approved kits, these kits fully comply with International Organization for Standardization (ISO) standards. Secondly, our study did not include all the NMPA-approved CT nucleic acid assay kits due to practical constraints. Thirdly, only nine laboratories participated in the external validation, which may restrict the broad applicability of our study results. However, we plan to conduct a nationwide EQA in the future to further evaluate the generalizability and robustness of the QCs.

Current sexually transmitted disease (STD) diagnosis kits typically test for CT, *Neisseria gonorrhoeae*, and *Ureaplasma urealyticum*. Thus, QCs that encompass a wide range of pathogens is crucial for enhancing nucleic acid detection in STDs. Additionally, different genotypes of CT result in varying disease severities, underscoring the need for QCs tailored to different genotypes to improve the diagnosis and treatment of CT infection (35). These considerations highlight a new direction for advancing CT QCs.

In conclusion, this study presents an innovative and practical method for QC preparation in CT NAATs. Additionally, it has the potential to provide a new idea for QC preparation in nucleic acid detection of intracellular parasitic pathogens.

## MATERIALS AND METHODS

### Plasmid construction and sgRNA design

**Fig 7:**
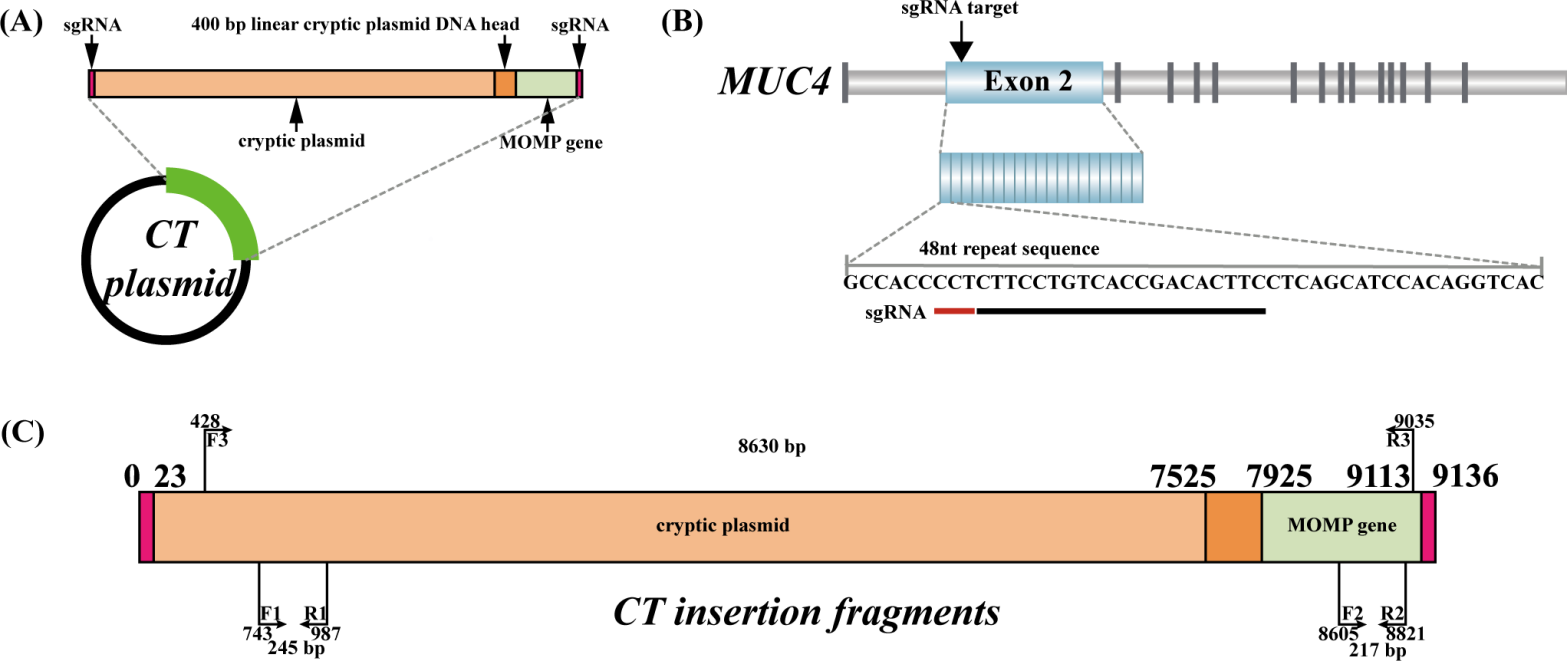
Illustration of experimental design. (**A**) CT plasmid mapping. The total length of the CT insertion sequence is 9,136 bp, including 23-bp sgRNA recognition sequence, 7,502 bp of the full length of the cryptic plasmid, 400 bp of the linear cryptic plasmid head, 1,188 bp of the full length of the *MOMP* gene, and 23 bp of the sgRNA recognition sequence. (**B**) Schematic representation of sgRNA targeting the 48-nt repeat tandem sequence in exon 2 of the *MUC4* gene. (**C**) Illustration of PCR-based screening primers for amplification.

We selected the full length of the cryptic plasmid (National Center for Biotechnology Information reference sequence: NC_001372.1) and the full length of the *MOMP* gene (GenBank: X52080.1) to construct the CT plasmid. The cryptic plasmid consists of circular DNA, whereas the fragment integrated into the cellular genome is linear DNA. To ensure that the designed primers at the head and tail of the linear fragment could effectively amplify the target sequences, a 400-bp linear cryptic plasmid DNA head was appended to the tail of the linear cryptic plasmid (Fig. 7A). The CT DNA sequence (Table S1) was synthesized by GenScript (Nanjing, China) and subsequently cloned into pUC57-Brick. Verification was conducted through sequencing and double digestion (Fig. S2A, B and S3).

To ensure the stable integration of the CT DNA sequence upon insertion into the HEK293T cell, we selected insertion sites that do not disrupt normal cell function. Considering the lengthy CT insertion sequence (exceeding 9 kb), we opted for a 48-nt repeat tandem sequence, consisting of approximately 400 copies located in exon 2 of the *MUC4* gene (Fig. 7B), for designing the sgRNA (44–46) (Table 2). The sgRNA-F and sgRNA-R (Table 2) were synthesized by Sangon Biotech (Shanghai, China). Following the previously established protocol (47), we cloned sgRNA oligonucleotides into pCAG-eCas9-green fluorescent protein (GFP)-U6-sgRNA (Addgene # 79145) and verified these by sequencing (Fig. S2C).

**TABLE 2:** Oligonucleotides used in this study^*a*^

Name	Sequences (5’→3’）	Intended use	Product length (bp)	Ref.
sgRNA	GAAGUGUCGGUGACAGGAAG	Directed cleavage of Cas9 protein	−	(44–46)
sgRNA-F	CACCGGAAGTGTCGGTGACAGGAAG	Annealed oligonucleotide	−	(44–46)
sgRNA-R	AAACCTTCCTGTCACCGACACTTCC	Annealed oligonucleotide	−	(44–46)
CT-F1	ACCATTTTTCCGGAGCGAG	Amplification of the 5′ end head of the CT insert sequence	245	−
CT-R1	AATGCCCGGGATTGGTTGAT			
CT-F2	ACGTTCTCTGTAACGCAGCA	Amplification of the 3′ end tail of the CT insertion sequence	217	−
CT-R2	TCAAAGCTTGCACGAGACCA			
CT-F3	ATTCCTCTAGTACAAACACCCAC	Amplification of the approximate full length of the CT insertion sequence	8,630	−
CT-R3	CAACTGTAACTGCGTATTTGTCT			
CT-F	CAGCTTGTAGTCCTGCTTGAGAGA	Amplification of CT gene in qPCR	109	(48, 49)
CT-R	CAAGAGTACATCGGTCAACGAAGA			
*GAPDH*-F	CCCCACACACATGCACTTACC	Amplification of *GAPDH* gene in qPCR	97	(50, 51)
*GAPDH*-R	CCTAGTCCCAGGGCTTTGATT			
CT Probe	FAM-CCCCACCATTTTTCCGGAGCGA-BHQ1	Probe for CT gene in qPCR	−	(48, 49)
*GAPDH* Probe	VIC-AAAGAGCTAGGAAGGACAGGCAACTTGGC-BHQ1	Probe for *GAPDH* gene in qPCR	−	(50, 51)

^*a*^ "−”, no PCR amplification product.

### Cell culture and transfection

The HEK293T cell line was chosen for its individual cell culture and high transfection efficiency. Obtained from the Cell Resource Center, Peking Union Medical College (PCRC, Beijing, China), HEK293T cells were cultured in DMEM (Thermo Fisher Scientific, Grand Island, NY, USA), supplemented with 10% fetal bovine serum (FBS, Thermo Fisher Scientific) and 1% penicillin-streptomycin (Thermo Fisher Scientific), at 37°C with 5% CO_2_. Cells were seeded at a density of 2 × 10^5^ cells/well in a 24-well plate (Corning, New York, USA). Upon reaching 70% confluency, cells were transfected with the Cas9/sgRNA plasmid and CT plasmid, utilizing Lipofectamine 3000 (Thermo Fisher Scientific, Carlsbad, CA, USA), following the manufacturer’s protocols. Following transfection, each well was replaced with fresh pre-warmed medium and incubated for another 48 hours.

### Monoclonal cell screening

After 48 hours post-transfection, cells were harvested and suspended in PBS (Cytiva, Massachusetts, USA) with 1% FBS and 2% penicillin-streptomycin for sorting. GFP-positive cells were individually sorted into 96-well plates (Corning) at the Peking Union Medical College State Key Laboratory of Medical Molecular Biology using a Sony MA900 cell sorter (Sony Biotechnology, San Jose, CA, USA). The sorted cells were cultured in DMEM supplemented with 20% FBS and 2% penicillin-streptomycin, at 37°C with 5% CO_2_. After 2–3 weeks of cultivation, they were expanded into 24-well plates.

To identify positive clones integrating the CT sequence, we designed different primer combinations for monoclonal cell screening (Fig. 7C). The sequences of the primers are shown in Table 2. gDNA from monoclonal cells was extracted using Quick Extract DNA Extract (Epicentre, Madison, WI, USA). For the initial screening and confirmation, primers (CT-F1 and CT-R1) were used to amplify the head of the CT insertion sequence. Subsequently, for further screening, primers (CT-F2 and CT-R2) were employed to amplify the tail of the CT insertion sequence. Primers (CT-F3 and CT-R3), capable of amplifying the 8,630-bp insertion sequence, were used for the final confirmation of positive clones and validation of the insertion of the full length of the cryptic plasmid and the *MOMP* gene. The amplification products were analyzed by electrophoresis on 1% agarose gel.

To verify the complete insertion of CT sequence, we sequenced the PCR-amplified 8,630-bp CT sequence. Primers CT-F3 and CT-R3 (Fig. 7C) were used for PCR amplification of gDNA from positive clonal cell lines. The amplified products were then submitted to Sangon Biotech for sequencing verification (Fig. S2D and E). The sequencing primers are listed in Table S4.

### Determination of the insertion copy number

To quantify QC concentrations in the sample panel preparation, we assessed the copy number of CT insertions in positive clonal cells using a previously reported method (52). The experimental design is illustrated in Fig. 3A. The methodology is outlined as follows: standards were prepared by mixing PCR-amplified and purified CT gene with the *GAPDH* gene at varying molar ratios. Subsequently, we used RT-qPCR to detect the Ct values of the CT gene and *GAPDH* gene in the standards. A standard curve was then constructed, illustrating the relationship between the ΔCt of the CT gene and the *GAPDH* gene and the molecular molar logarithmic ratio between the two genes. The determination of the CT gene’s copy number in positive clonal cells was accomplished by using the measured ∆Ct to derive the molecular molar logarithmic ratio of the CT gene to the *GAPDH* gene from the standard curve.

The procedure was conducted as follows: gDNA was extracted from wild-type HEK293T cells, residual mixed pool cells after flow cytometric sorting, and positive clonal cells (C12) using the QIAamp DNA Mini Kit (QIAGEN, Hilden, Germany). The CT gene was amplified from the gDNA of the residual mixed pool cells using the CT-F and CT-R primers (48, 49). Simultaneously, amplification of the *GAPDH* gene from the gDNA of wild-type HEK293T cells was performed using the *GAPDH*-F and *GAPDH*-R primers (50, 51). The amplification products of CT and *GAPDH* genes were purified using the MinElute PCR Purification Kit (QIAGEN), and their concentrations were determined by Qubit (version 3.0) (Thermo Fisher Scientific, Waltham, USA). Molar concentrations were calculated based on the relative molecular mass. To ensure quantification precision, the purified CT and *GAPDH* genes were diluted with Tris-EDTA buffer (Thermo Fisher Scientific), aiming for Ct values between 20 and 30 cycles. Standards were prepared by mixing the diluted samples at different molar ratios. Subsequently, standards and gDNA from positive clone cells were analyzed using the previously mentioned primers (CT-F/CT-R and *GAPDH*-F/*GAPDH*-R) and probes (CT and *GAPDH*). Ct values were determined using the Applied Biosystems 7500 Real-Time PCR Systems (Thermo Fisher Scientific, Waltham, USA). The PCR system includes 10 µL of TaqMan Gene Expression Master Mix (2×) (Thermo Fisher Scientific), 0.6 µL of 10-µM forward and reverse primers, 0.2 µL of 10-µM CT and *GAPDH* probes, 1 µL of gDNA, and nuclease-free water to make up a 20-µL reaction system. Amplification conditions were as follows: 50°C for 2 min, 95°C for 10 min, 40 cycles of 95°C for 15 s, and 60°C for 60 s (signal acquisition). Each sample was tested three times under replicate conditions.

### Optimization of cellular quality control materials preservation solutions

PEG and methanol are commonly used cell preservation solutions in various applications, including nucleic acid amplification (38, 53). However, we observed potential inhibition of PCR amplification by these components (41, 42). Therefore, we optimized the preservation protocol for cellular QCs: cells were initially treated with a preservation solution containing PEG and methanol. Subsequently, centrifugation removed these components, and the cells were resuspended in PBS. Two control groups (PBS and DMEM groups) and two experimental groups (preservation solution and optimization groups) were established. The preservation solution consisted of 10% (vol/vol) PEG 300 (Sigma-Aldrich, St. Louis, USA) and 90% (vol/vol) methanol (Sigma-Aldrich) (38). For cell dilutions, 0.9-g/L sodium azide (Sigma-Aldrich) was added to the PBS and DMEM.

The procedure for the PBS, DMEM, and preservation solution groups was as follows: after trypsin (Thermo Fisher Scientific) digestion, cells were centrifuged at 157 × *g* for 5 min (Eppendorf centrifuge, model 5702, rotor A-4–38; Eppendorf, USA), and the supernatant was removed. Subsequently, PBS, DMEM, or preservation solution was added to the cell sediment to achieve the desired concentration. The procedure for the optimization group was as follows: approximately 1 × 10^7^ cells of positive clonal cells were digested with trypsin, centrifuged at 157 × *g* for 5 min (Eppendorf centrifuge, model 5702, rotor A-4–38), and the supernatant was removed. Then, 3.5 mL of cell preservation solution was added to the cell precipitate. The cell suspension was gently mixed and processed for 5 min at room temperature. After removing the supernatant through centrifugation at 18,514 × *g* for 1 min (Eppendorf centrifuge, model 5810R, rotor F34-6-38), two washes were performed using 7 mL of PBS solution (without sodium azide) by centrifugation at 157 × *g* for 5 min (Eppendorf centrifuge, model 5702, rotor A-4–38). Finally, the cells were diluted to the appropriate concentration with PBS (0.9-g/L sodium azide).

To investigate efficient cell preservation protocols, we conducted a stability pre-experiment. Following the outlined procedure, we prepared four sets of QCs at a concentration of 10^5^ cells/mL in a volume of 1 mL and performed preliminary stability assessments at 37°C at the time points of 0 day and 1 and 2 weeks.

The CT nucleic acid assay kit (Hybribio, Guangzhou, China), approved by the NMPA, was used to analyze the nucleic acids of the QCs. The nucleic acids of the QCs were extracted using the QIAamp DNA Mini kit (QIAGEN), along with the extraction reagents provided in the assay kit (Hybribio). Ct values were determined using the Applied Biosystems 7500 Real-Time PCR Systems (Thermo Fisher Scientific). The sample processing procedure was conducted as follows: the QCs were centrifuged at 15,493 × *g* for 1 min (Eppendorf centrifuge, model 5424, rotor F-45–24-11), and the supernatant was discarded. Subsequently, 50 µL of cell lysis buffer was added to the sample and shaken well to ensure complete resuspension of the cells, followed by boiling the mixture for 10 min. Finally, the QCs were centrifuged at 15,493 × *g* for 10 min (Eppendorf centrifuge, model 5424, rotor F-45–24-11), and the supernatant was retained for spare use. The assay procedure was conducted as follows: the PCR system included 17.5 µL of PCR Mix, 0.5 µL of DNA polymerase, and 2 µL of templates to make up a 20-µL reaction system. Amplification conditions were as follows: 95°C for 10 min, 45 cycles of 95°C for 15 s and 60°C for 60 s (signal acquisition), and 38°C for 5 s.

Following preliminary stability assessment results, the PBS group and the optimization group were chosen for freeze-thaw stability assessments. The freeze-thaw processing procedure was conducted as follows: The frozen QCs were thawed at room temperature for 30 min and subsequently frozen in a refrigerator at −20°C for 1 hour to complete a freeze-thaw cycle. The nucleic acids of QCs, extracted in the freeze-thaw stability experiment, were detected using the Hybribio assay kit. Data were analyzed through linear trend analysis.

### Preparation of quality control materials

We generated 100 sets of three QCs in a volume of 1 mL. Each set included 1 × 10^5^ cells/mL of 293T-negative QCs, 1 × 10^4^ cells/mL of CT-weak-positive QCs, and 1 × 10^5^ cells/mL of CT-moderate-positive QCs. These sets were prepared using the optimization group’s protocol for subsequent QC evaluation experiments.

The prepared QCs were exposed to specific experimental conditions as needed. Any excess QCs were then stored at −80°C.

### Assessment of homogeneity and stability of quality control materials

To assess QCs homogeneity and stability, we determined the Ct values of the CT-moderate-positive QCs using the Daan Gene assay kit (Guangzhou, China), approved by the NMPA. The gDNA from the CT-moderate-positive QCs was extracted with the QIAamp DNA Mini Kit. Ct values were measured on the Applied Biosystems 7500 Real-Time PCR Systems. The assay procedure was conducted as follows: The PCR system includes 17 µL of CT PCR reaction solution A, 3 µL of CT PCR reaction solution B, and 5 µL of templates to make up a 25-µL reaction system. Amplification conditions were as follows: 50°C for 2 min, 95°C for 15 min, and 40 cycles of 94°C for 15 s and 55°C for 45 s (signal acquisition).

The study design followed the guidelines outlined in ISO Guides 35:2017 (54), JJF1343-2022 (55), and CNAS-GL032 2018 (56).

#### Homogeneity verification

We randomly selected 10 out of 100 QCs for validation, and each sample was tested three times under repetitive conditions. Data were assessed using one-way ANOVA.

#### Stability verification

Long-term stability tests were conducted at −20°C, 4°C, 25°C, and 37°C, with time points of 1, 3, 7, 14, 30, and 60 days. Additionally, thermal stability tests were performed at 43°C with time points at 1, 3, 7, and 14 days. At each time point, except for time zero, samples were removed from storage temperature and processed according to the established protocol. For the zero time point, the average value from the homogeneity study was used. Each sample underwent three repetitions under consistent conditions, and the data were analyzed using linear trend analysis.

### Validation of commutability in quality control materials

#### Internal commutability validation

To ensure consistent and comparable QCs performance across various commercial kits, we conducted an internal applicability validation (57–59). To date, the NMPA in China has approved 19 CT nucleic acid test kits, predominantly utilizing TaqMan qPCR assays (https://www.nmpa.gov.cn/datasearch/home-index.html#category=ylqx). For internal commutability evaluation, we selected eight widely used kits in China, all based on the TaqMan qPCR. These kits included TIANLONG (approval no. 20153400366), Daan Gene (approval no. 20163401027), Hybribio (approval no. 20143401937), ACON Biotech (approval no. 20143402231), Sansure Biotech Inc (approval no. 20153400084), QIAGEN (approval no. 20163400008), Daan Gene (approval no. 20213400572), and BioPerfectus Technologies (approval no. 20183400058).

We extracted and detected nucleic acids from CT-moderate-positive QCs according to the kit instructions, utilizing Applied Biosystems 7500 Real-Time PCR Systems. Detailed procedures are available in the supplemental materials.

#### External commutability validation

To validate the practical utility of QCs in laboratory testing, external applicability validation was conducted (57–59). Sample panels were prepared with 1-mL volumes, comprising 10^5^ cells/mL of 293T-negative QCs, as well as 10^4^ and 10^5^ cells/mL of CT-weak-positive QCs and CT-moderate-positive QCs. Subsequently, we employed the CT nucleic acid detection kit to assess the validity of the negative, weak-positive, and moderate-positive QCs in the sample panels. After completing the validity assay, the QCs were shipped as blinded samples at room temperature to nine laboratories across various regions in China, including hospital laboratories, third-party test laboratories, and reagent companies. Each laboratory processed, tested, and analyzed the QCs following its standard workflow, utilizing its instrumentation, sample handling procedure, and assay kit.

### Statistical analysis

The collected data were organized in Microsoft Excel (version 16.72). One-way ANOVA was performed using SPSS (version 26.0). Linear trend analysis and plotting were conducted with GraphPad Prism 9 (version 9.5.1). A *P* value of <0.05 was considered statistically significant.

## SUPPLEMENTAL MATERIAL

Supplemental material spectrum.00837-24-s0001.docx Tables S1 to S4; Fig. S1 to S3.

ASM does not own the copyrights to Supplemental Material that may be linked to, or accessed through, an article. The authors have granted ASM a non-exclusive, world-wide license to publish the Supplemental Material files. Please contact the corresponding author directly for reuse.
