# Supplementary material for: A novel method for the preparation of reproducible, stable, and non-infectious quality control materials for Chlamydia trachomatis nucleic acid detection
Source: Microbiol Spectr. 2024 Oct 7;12(11):e00837-24. doi: 10.1128/spectrum.00837-24 (PMC11536990; doi:10.1128/spectrum.00837-24)
Supplement: Supplemental material — Tables S1 to S4; Fig. S1 to S3. [file spectrum.00837-24-s0001.docx]

**Supplementary materials**

A novel method for the preparation of reproducible, stable, and non-infectious quality control materials for Chlamydia trachomatis nucleic acid detection

Jing Li,^a,b^ Kuo Zhang,^a,b,c^ Yanxi Han,^a,b,c^ Rongxue Peng,^a,b,c^ Lin Li,^a,b,c^ Jinming Li,^a,b,c^# Guigao Lin^a,b,c^ #

^a^ National Center for Clinical Laboratories, Institute of Geriatric Medicine, Chinese Academy of Medical Sciences, Beijing Hospital/ National Center of Gerontology, Beijing, China

^b^ Graduate School, Chinese Academy of Medical Sciences & Peking Union Medical College, Beijing, China

^c^ Beijing Engineering Research Center of Laboratory Medicine, Beijing, China

#Address correspondence to Jinming Li, [jmli@nccl.org.cn](mailto:jmli@nccl.org.cn) or Guigao Lin, [gglin@nccl.org.cn](mailto:gglin@nccl.org.cn).

**This file includes：**

Detailed procedures for different kits

Tables S1-S4

Figures S1-S3

**Detailed procedures for different kits**

***TIANLONG (Suzhou, China) (Approval No. 20153400366)***

The sample processing procedure was conducted as follows: The QCs were centrifuged at 13,201 ×g for 5 minutes (Eppendorf centrifuge, model 5424, rotor F-45-24-11, USA), and the supernatant was discarded. Subsequently, 50 μL of nucleic acid extraction solution was added, thoroughly mixed, and centrifuged at 367 ×g for 10 seconds (Eppendorf centrifuge, model 5424, rotor F-45-24-11, USA). The samples were then placed in a water bath at 100°C for 10 minutes. Finally, the QCs were centrifuged at 13,201 ×g for 10 minutes (Eppendorf centrifuge, model 5424, rotor F-45-24-11, USA), and the supernatant was retained for spare use.

The assay procedure was conducted as follows: The PCR system included 35.2 μL of CT reaction mixture, 0.8 μL of Taq enzyme, and 4 μL of templates to make up a 40 μL reaction system. Amplification conditions were as follows: 95°C for 3 min; 40 cycles of 94°C for 15s and 60°C for 30s (signal acquisition).

***DAAN GENE (Guangzhou, China) (Approval No. 20163401027)***

The sample processing procedure was conducted as follows: The QCs were centrifuged at 13,201 ×g for 5 minutes (Eppendorf centrifuge, model 5424, rotor F-45-24-11, USA), and the supernatant was discarded. Subsequently, 50 μL of DNA extraction solution was added, ensuring thorough mixing, and they were incubated in a boiling water bath for 10 minutes. The samples were then transferred to 4°C and allowed to stand for 6-8 hours to ensure adequate lysis. Finally, the QCs were centrifuged at 13,201 ×g for 2 minutes (Eppendorf centrifuge, model 5424, rotor F-45-24-11, USA), and the supernatant was retained for future use.

The assay procedure was conducted as follows: The PCR system included 40 μL of CT-PCR reaction solution, 3 μL of Taq enzyme, and 2 μL of templates to make up a 45 μL reaction system. Amplification conditions were as follows: 93°C for 2 min; 10 cycles of 93°C for 45s and 55°C for 60s; 30 cycles of 93°C for 30s and 55°C for 45s (signal acquisition).

***Hybribio (Guangzhou, China) (Approval No. 20143401937)***

The sample processing procedure was conducted as follows: The QCs were centrifuged at 15,493 ×g for 1 minute (Eppendorf centrifuge, model 5424, rotor F-45-24-11, USA), and the supernatant was discarded. Subsequently, 50 μL of cell lysis buffer was added to the sample and shaken well to ensure complete resuspension of the cells, followed by boiling the mixture for 10 minutes. Finally, the QCs were centrifuged at 15,493 ×g for 10 minutes (Eppendorf centrifuge, model 5424, rotor F-45-24-11, USA), and the supernatant was retained for spare use.

The assay procedure was conducted as follows: The PCR system included 17.5 μL of PCR Mix, 0.5 μL of DNA polymerase, and 2 μL of templates to make up a 20 μL reaction system. Amplification conditions were as follows: 95°C for 10 min; 45 cycles of 95°C for 15s and 60°C for 60s (signal acquisition); and 38°C for 5s.

***ACON BIOTECH (Hangzhou, China) (Approval No. 20143402231)***

The sample processing procedure was conducted as follows: The QCs were centrifuged at 13,201 ×g for 5 minutes (Eppendorf centrifuge, model 5424, rotor F-45-24-11, USA), and the supernatant was discarded. Subsequently, 50 μL of nucleic acid extraction solution was added, thoroughly mixed, and centrifuged at 367 ×g for 10 seconds (Eppendorf centrifuge, model 5424, rotor F-45-24-11, USA). The sample was then placed in a water bath at 100°C for 10 minutes. Finally, the QCs were centrifuged at 13,201 ×g for 10 minutes (Eppendorf centrifuge, model 5424, rotor F-45-24-11, USA), and the supernatant was retained for spare use.

The assay procedure was conducted as follows: The PCR system included 35.6 μL of CT reaction solution, 0.4 μL of Taq enzyme, and 4 μL of templates to make up a 40 μL reaction system. Amplification conditions were as follows: 95°C for 3 min; 40 cycles of 94°C for 15s and 60°C for 30s (signal acquisition).

***Sansure Biotech Inc (Changsha, China) (Approval No. 20153400084)***

The sample processing procedure 1 was conducted as follows: The QCs were centrifuged at 13,201 ×g for 5 minutes (Eppendorf centrifuge, model 5424, rotor F-45-24-11, USA), and the supernatant was discarded. Subsequently, 50 μL of sample release agent was added, and the mixture was pipetted and mixed as a sample to be tested.

The sample processing procedure 2 was conducted as follows: The 5 μL of QCs were added to 5 μL of sample release agent and mixed by pipetting 3-5 times with intervals of 10 minutes or more, then set aside.

The assay procedure was conducted as follows: The PCR system included 38 μL of PCR reaction solution, 2 μL of enzyme mixture, 1 μL of internal standard, and 10 μL of templates to make up a 51 μL reaction system. Amplification conditions were as follows: 50°C for 2 min; 94°C for 5 min; 45 cycles of 94°C for 15s and 57°C for 30s (signal acquisition); and 50°C for 2 min.

***QIAGEN (Shenzhen, China) (Approval No. 20163400008)***

The sample processing procedure was conducted as follows: The QCs were centrifuged at 15,000 ×g for 10 minutes (Eppendorf centrifuge, model 5424, rotor F-45-24-11, USA), and the supernatant was discarded. Then, 50 μL of DNA extraction solution 2 was added, mixed, and centrifuged at 2000 ×g for 10 seconds (Eppendorf centrifuge, model 5424, rotor F-45-24-11, USA). The sample was subsequently incubated in a water bath at 100°C for 10 minutes. Finally, the QCs were centrifuged at 15,000 ×g for 5 minutes (Eppendorf centrifuge, model 5424, rotor F-45-24-11, USA), and the supernatant was retained for spare use.

The assay procedure was conducted as follows: The PCR system included 33.3 μL of PCR reaction solution, 0.7 μL of HS-Taq Plus enzyme, 0.06 μL of UNG and 6 μL of templates to make up a 40 μL reaction system. Amplification conditions were as follows: 37°C for 5 min; 95°C for 5 min; 40 cycles of 95°C for 15s and 60°C for 40s (signal acquisition).

***DAAN GENE (Guangzhou, China) (Approval No. 20213400572)***

The assay procedure was conducted as follows: The PCR system includes 17 μL of CT PCR reaction solution A, 3 μL of CT PCR reaction solution B, and 5 μL of templates to make up a 25 μL reaction system. Amplification conditions were as follows: 50°C for 2 min; 95°C for 15 min; 40 cycles of 94°C for 15s and 55°C for 45s (signal acquisition).

***BioPerfectus technologies (Taizhou, China) (Approval No. 20183400058)***

The assay procedure was conducted as follows: The PCR system includes 18 μL of CT reaction solution, 2 μL of enzyme mixture, and 5 μL of templates to make up a 25 μL reaction system. Amplification conditions were as follows: 37°C for 5 min; 95°C for 5 min; 40 cycles of 95°C for 10s and 55°C for 45s (signal acquisition).

**TABLES**

**TABLE S1** The sequence of CT DNA.

| Name | Sequence（5’→3’）* |
| --- | --- |
| CT DNA | CCTCTTCCTGTCACCGACACTTCGGATCCGTAAGTTAGACGAAATTTTGTCTTTGCGCACAGACGATCTATTTTTTGCATCCAATCAGATTTCCTTTCGCATTAAAAAAAGACAGAATAAAGAAACCAAAATTCTAATCACATTTCCTATCAGCTTAATGGAAGAGTTGCAAAAATACACTTGTGGGAGAAATGGGAGAGTATTTGTTTCTAAAATAGGGATTCCTGTAACAACAAGTCAGGTTGCGCATAATTTTAGGCTTGCAGAGTTCCATAGTGCTATGAAAATAAAAATTACTCCCAGAGTACTTCGTGCAAGCGCTTTGATTCATTTAAAGCAAATAGGATTAAAAGATGAGGAAATCATGCGTATTTCCTGTCTTTCATCGAGACAAAGTGTGTGTTCTTATTGTTCTGGGGAAGAGGTAATTCCTCTAGTACAAACACCCACAATATTGTGATATAATTAAAATTATATTCATATTCTGTTGCCAGAAAAAACACCTTTAGGCTATATTAGAGCCATCTTCTTTGAAGCGTTGTCTTCTCGAGAAGATTTATCGTACGCAAATATCATCTTTGCGGTTGCGTGTCCTGTGACCTTCATTATGTCGGAGTCTGAGCACCCTAGGCGTTTGTACTCCGTCACAGCGGTTGCTCGAAGCACGTGCGGGGTTATTTTAAAAGGGATTGCAGCTTGTAGTCCTGCTTGAGAGAACGTGCGGGCGATTTGCCTTAACCCCACCATTTTTCCGGAGCGAGTTACGAAGACAAAACCTCTTCGTTGACCGATGTACTCTTGTAGAAAGTGCATAAACTTCTGAGGATAAGTTATAATAATCCTCTTTTCTGTCTGACGGTTCTTAAGCTGGGAGAAAGAAATGGTAGCTTGTTGGAAACAAATCTGACTAATCTCCAAGCTTAAGACTTCAGAGGAGCGTTTACCTCCTTGGAGCATTGTCTGGGCGATCAACCAATCCCGGGCATTGATTTTTTTTAGCTCTTTTAGGAAGGATGCTGTTTGCAAACTGTTCATCGCATCCGTTTTTACTATTTCCCTGGTTTTAAAAAATGTTCGACTATTTTCTTGTTTAGAAGGTTGCGCTATAGCGACTATTCCTTGAGTCATCCTGTTTAGGAATCTTGTTAAGGAAATATAGCTTGCTGCTCGAACTTGTTTAGTACCTTCGGTCCAAGAAGTCTTGGCAGAGGAAACTTTTTTAATCGCATCTAGGATTAGATTATGATTTAAAAGGGAAAACTCTTGCAGATTCATATCCAAGGACAATAGACCAATCTTTTCTAAAGACAAAAAAGATCCTCGATATGATCTACAAGTATGTTTGTTGAGTGATGCGGTCCAATGCATAATAACTTCGAATAAGGAGAAGCTTTTCATGCGTTTCCAATAGGATTCTTGGCGAATTTTTAAAACTTCCTGATAAGACTTTTCACTATATTCTAACGACATTTCTTGCTGCAAAGATAAAATCCCTTTACCCATGAAATCCCTCGTGATATAACCTATCCGTAAAATGTCCTGATTAGTGAAATAATCAGGTTGTTAACAGGATAGCACGCTCGGTATTTTTTTATATAAACATGAAAACTCGTTCCGAAATAGAAAATCGCATGCAAGATATCGAGTATGCGTTGTTAGGTAAAGCTCTGATATTTGAAGACTCTACTGAGTATATTCTGAGGCAGCTTGCTAATTATGAGTTTAAGTGTTCTCATCATAAAAACATATTCATAGTATTTAAACACTTAAAAGACAATGGATTACCTATAACTGTAGACTCGGCTTGGGAAGAGCTTTTGCGGCGTCGTATCAAAGATATGGACAAATCGTATCTCGGGTTAATGTTGCATGATGCTTTATCAAATGACAAGCTTAGATCCGTTTCTCATACGGTTTTCCTCGATGATTTGAGCGTGTGTAGCGCTGAAGAAAATTTGAGTAATTTCATTTTCCGCTCGTTTAATGAGTACAATGAAAATCCATTGCGTAGATCTCCGTTTCTATTGCTTGAGCGTATAAAGGGAAGGCTTGATAGTGCTATAGCAAAGACTTTTTCTATTCGCAGCGCTAGAGGCCGGTCTATTTATGATATATTCTCACAGTCAGAAATTGGAGTGCTGGCTCGTATAAAAAAAAGACGAGTAGCGTTCTCTGAGAATCAAAATTCTTTCTTTGATGGCTTCCCAACAGGATACAAGGATATTGATGATAAAGGAGTTATCTTAGCTAAAGGTAATTTCGTGATTATAGCAGCTAGACCATCTATAGGGAAAACAGCTTTAGCTATAGACATGGCGATAAATCTTGCGGTTACTCAACAGCGTAGAGTTGGTTTCCTATCTCTAGAAATGAGCGCAGGTCAAATTGTTGAGCGGATTATTGCTAATTTAACAGGAATATCTGGTGAAAAATTACAAAGAGGGGATCTCTCTAAAGAAGAATTATTCCGAGTAGAAGAAGCTGGAGAAACGGTTAGAGAATCACATTTTTATATCTGCAGTGATAGTCAGTATAAGCTTAACTTAATCGCGAATCAGATCCGGTTGCTGAGAAAAGAAGATCGAGTAGACGTAATATTTATCGATTACTTGCAGTTGATCAACTCATCGGTTGGAGAAAATCGTCAAAATGAAATAGCAGATATATCTAGAACCTTAAGAGGTTTAGCCTCAGAGCTAAACATTCCTATAGTTTGTTTATCCCAACTATCTAGAAAAGTTGAGGATAGAGCAAATAAAGTTCCCATGCTTTCAGATTTGCGAGACAGCGGTCAAATAGAGCAAGACGCAGATGTGATTTTGTTTATCAATAGGAAGGAATCGTCTTCTAATTGTGAGATAACTGTTGGGAAAAATAGACATGGATCGGTTTTCTCTTCGGTATTACATTTCGATCCAAAAATTAGTAAATTCTCCGCTATTAAAAAAGTATGGTAAATTATAGTAACTGCCACTTCATCAAAAGTCCTATCCACCTTGAAAATCAGAAGTTTGGAAGAAGACCTGGTCAATCTATTAAGATATCTCCCAAATTGGCTCAAAATGGGATGGTAGAAGTTATAGGTCTTGATTTTCTTTCATCTCATTACCATGCATTAGCAGCTATCCAAAGATTACTGACCGCAACGAATTACAAGGGGAACACAAAAGGGGTTGTTTTATCCAGAGAATCAAATAGTTTTCAATTTGAAGGATGGATACCAAGAATCCGTTTTACAAAAACTGAATTCTTAGAGGCTTATGGAGTTAAGCGGTATAAAACATCCAGAAATAAGTATGAGTTTAGTGGAAAAGAAGCTGAAACTGCTTTAGAAGCCTTATACCATTTAGGACATCAACCGTTTTTAATAGTGGCAACTAGAACTCGATGGACTAATGGAACACAAATAGTAGACCGTTACCAAACTCTTTCTCCGATCATTAGGATTTACGAAGGATGGGAAGGTTTAACTGACGAAGAAAATATAGATATAGACTTAACACCTTTTAATTCACCACCTACACGGAAACATAAAGGGTTCGTTGTAGAGCCATGTCCTATCTTGGTAGATCAAATAGAATCCTACTTTGTAATCAAGCCTGCAAATGTATACCAAGAAATAAAAATGCGTTTCCCAAATGCATCAAAGTATGCTTACACATTTATCGACTGGGTGATTACAGCAGCTGCGAAAAAGAGACGAAAATTAACTAAGGATAATTCTTGGCCAGAAAACTTGTTATTAAACGTTAACGTTAAAAGTCTTGCATATATTTTAAGGATGAATCGGTACATCTGTACAAGGAACTGGAAAAAAATCGAGTTAGCTATCGATAAATGTATAGAAATCGCCATTCAGCTTGGCTGGTTATCTAGAAGAAAACGCATTGAATTTCTGGATTCTTCTAAACTCTCTAAAAAAGAAATTCTATATCTAAATAAAGAGCGCTTTGAAGAAATAACTAAGAAATCTAAAGAACAAATGGAACAATTAGAACAAGAATCTATTAATTAATAGCAAGCTTGAAACTAAAAACCTAATTTATTTAAAGCTCAAAATAAAAAAGAGTTTTAAAATGGGAAATTCTGGTTTTTATTTGTATAACACTGAAAACTGCGTCTTTGCTGATAATATCAAAGTTGGGCAAATGACAGAGCCGCTCAAGGACCAGCAAATAATCCTTGGGACAACATCAACACCTGTCGCAGCCAAAATGACAGCTTCTGATGGAATATCTTTAACAGTCTCCAATAATTCATCAACCAATGCTTCTATTACAATTGGTTTGGATGCGGAAAAAGCTTACCAGCTTATTCTAGAAAAGTTGGGAGATCAAATTCTTGATGGAATTGCTGATACTATTGTTGATAGTACAGTCCAAGATATTTTAGACAAAATCAAAACAGACCCTTCTCTAGGTTTGTTGAAAGCTTTTAACAACTTTCCAATCACTAATAAAATTCAATGCAACGGGTTATTCACTCCCAGTAACATTGAAACTTTATTAGGAGGAACTGAAATAGGAAAATTCACAGTCACACCCAAAAGCTCTGGGAGCATGTTCTTAGTCTCAGCAGATATTATTGCATCAAGAATGGAAGGCGGCGTTGTTCTAGCTTTGGTACGAGAAGGTGATTCTAAGCCCTGCGCGATTAGTTATGGATACTCATCAGGCATTCCTAATTTATGTAGTCTAAGAACCAGTATTACTAATACAGGATTGACTCCGACAACGTATTCATTACGTGTAGGCGGTTTAGAAAGCGGTGTGGTATGGGTTAATGCCCTTTCTAATGGCAATGATATTTTAGGAATAACAAATACTTCTAATGTATCTTTTTTAGAGGTAATACCTCAAACAAACGCTTAAACAATTTTTATTGGATTTTTCTTATAGGTTTTATATTTAGAGAAAACAGTTCGAATTACGGGGTTTGTTATGCAAAATAAAAGAAAAGTGAGGGACGATTTTATTAAAATTGTTAAAGATGTGAAAAAAGATTTCCCCGAATTAGACCTAAAAATACGAGTAAACAAGGAAAAAGTAACTTTCTTAAATTCTCCCTTAGAACTCTACCATAAAAGTGTCTCACTAATTCTAGGACTGCTTCAACAAATAGAAAACTCTTTAGGATTATTCCCAGACTCTCCTGTTCTTGAAAAATTAGAGGATAACAGTTTAAAGCTAAAAAAGGCTTTGATTATGCTTATCTTGTCTAGAAAAGACATGTTTTCCAAGGCTGAATAGACAACTTACTCTAACGTTGGAGTTGATTTGCACACCTTAGTTTTTTGCTCTTTTAAGGGAGGAACTGGAAAAACAACACTTTCTCTAAACGTGGGATGCAACTTGGCCCAATTTTTAGGGAAAAAAGTGTTACTTGCTGACCTAGACCCGCAATCCAATTTATCTTCTGGATTGGGGGCTAGTGTCAGAAGTGACCAAAAAGGCTTGCACGACATAGTATACACATCAAACGATTTAAAATCAATCATTTGCGAAACAAAAAAAGATAGTGTGGACCTAATTCCTGCATCATTTTCATCCGAACAGTTTAGAGAATTGGATATTCATAGAGGACCTAGTAACAACTTAAAGTTATTTCTGAATGAGTACTGCGCTCCTTTTTATGACATCTGCATAATAGACACTCCACCTAGCCTAGGAGGGTTAACGAAAGAAGCTTTTGTTGCAGGAGACAAATTAATTGCTTGTTTAACTCCAGAACCTTTTTCTATTCTAGGGTTACAAAAGATACGTGAATTCTTAAGTTCGGTCGGAAAACCTGAAGAAGAACACATTCTTGGAATAGCTTTGTCTTTTTGGGATGATCGTAACTCGACTAACCAAATGTATATAGACATTATCGAGTCTATTTACAAAAACAAGCTTTTTTCAACAAAAATTCGTCGAGATATTTCTCTCAGCCGTTCTCTTCTTAAAGAAGATTCTGTAGCTAATGTCTATCCAAATTCTAGGGCCGCAGAAGATATTCTGAAGTTAACGCATGAAATAGCAAATATTTTGCATATCGAATATGAACGAGATTACTCTCAGAGGACAACGTGAACAAACTAAAAAAAGAAGCGGATGTCTTTTTTAAAAAAAATCAAACTGCCGCTTCTCTAGATTTTAAGAAGACGCTTCCCTCCATTGAACTATTCTCAGCAACTTTGAATTCTGAGGAAAGTCAGAGTTTGGATCGATTATTTTTATCAGAGTCCCAAAACTATTCGGATGAAGAATTTTATCAAGAAGACATCCTAGCGGTAAAACTGCTTACTGGTCAGATAAAATCCATACAGAAGCAACACGTACTTCTTTTAGGAGAAAAAATCTATAATGCTAGAAAAATCCTGAGTAAGGATCACTTCTCCTCAACAACTTTTTCATCTTGGATAGAGTTAGTTTTTAGAACTAAGTCTTCTGCTTACAATGCTCTTGCATATTACGAGCTTTTTATAAACCTCCCCAACCAAACTCTACAAAAAGAGTTTCAATCGATCCCCTATAAATCCGCATATATTTTGGCCGCTAGAAAAGGCGATTTAAAAACCAAGGTCGATGTGATAGGGAAAGTATGTGGAATGTCGAACTCATCGGCGATAAGGGTGTTGGATCAATTTCTTCCTTCATCTAGAAACAAAGACGTTAGAGAAACGATAGATAAGTCTGATTCAGAGAAGAATCGCCAATTATCTGATTTCTTAATAGAGATACTTCGCATCATGTGTTCCGGAGTTTCTTTGTCCTCCTATAACGAAAATCTTCTACAACAGCTTTTTGAACTTTTTAAGCAAAAGAGCTGATCCTCCGTCAGCTCATATATATATATCTATTATATATATATATTTAGGGATTTGATTTCACGAGAGAGATTTGCAACTCTTGGTGGTAGACTTTGCAACTCTTGGTGGTAGACTTTGCAACTCTTGGTGGTAGACTTTGCAACTCTTGGTGGTAGACTTGGTCATAATGGACTTTTGTTAAAAAATTTATTAAAATCTTAGAGCTCCGATTTTGAATAGCTTTGGTTAAGAAAATGGGCTCGATGGCTTTCCATAAAAGTAGATTGTTTTTAACTTTTGGGGACGCGTCGGAAATTTGGTTATCTACTTTATCTTATCTAACTAGAAAAAATTATGCGTCTGGGATTAACTTTCTTGTTTCTTTAGAGATTCTGGATTTATCGGAAACCTTGATAAAGGCTATTTCTCTTGACCACAGCGAATCTTTGTTTAAAATCAAGTCTCTAGATGTTTTTAATGGAAAAGTTGTTTCAGAGGCATCTAAACAGGCTAGAGCGGCATGCTACATATCTTTCACAAAGTTTTTGTATAGATTGACCAAGGGATATATTAAACCCGCTATTCCATTGAAAGATTTTGGAAACACTACATTTTTTAAAATCCGAGACAAAATCAAAACAGAATCGATTTCTAAGCAGGAATGGACAGTTTTTTTTGAAGCGCTCCGGATAGTGAATTATAGAGACTATTTAATCGGTAAATTGATTGTACAAGGGATCCGTAAGTTAGACGAAATTTTGTCTTTGCGCACAGACGATCTATTTTTTGCATCCAATCAGATTTCCTTTCGCATTAAAAAAAGACAGAATAAAGAAACCAAAATTCTAATCACATTTCCTATCAGCTTAATGGAAGAGTTGCAAAAATACACTTGTGGGAGAAATGGGAGAGTATTTGTTTCTAAAATAGGGATTCCTGTAACAACAAGTCAGGTTGCGCATAATTTTAGGCTTGCAGAGTTCCATAGTGCTATGAAAATAAAAATTACTCCCAGAGTACTTCGTGCAAGCGCTTTGATTCATTTAAAGCAAATAGGATTAAAAGATGAGGAAATCATGCGTATTTCCTGTCTTTCATCGAGACAAAGTGTGTGTTCTTATTGTTCTGGGGAAGAATGAAAAAACTCTTGAAATCGGTATTAGTATTTGCCGCTTTGAGTTCTGCTTCCTCCTTGCAAGCTCTGCCTGTGGGGAATCCTGCTGAACCAAGCCTTATGATCGACGGAATTCTGTGGGAAGGTTTCGGCGGAGATCCTTGCGATCCTTGCACCACTTGGTGTGACGCTATCAGCATGCGTATGGGTTACTATGGTGACTTTGTTTTCGACCGTGTTTTGAAAACAGATGTGAATAAAGAGTTTGAAATGGGCGAGGCTTTAGCCGGAGCTTCTGGGAATACGACCTCTACTCTTTCAAAATTGGTAGAACGAACGAACCCTGCATATGGCAAGCATATGCAAGACGCAGAGATGTTTACCAATGCCGCTTGCATGACATTGAATATTTGGGATCGTTTTGATGTATTCTGTACATTAGGAGCCACCAGTGGATATCTTAAAGGAAATTCAGCATCTTTCAACTTAGTTGGGTTATTCGGCGATGGTGTAAACGCCACGAAACCTGCTGCAGATAGTATTCCTAACGTGCAGTTAAATCAGTCTGTGGTGGAACTGTATACAGATACTACTTTTGCTTGGAGTGTTGGAGCTCGTGCAGCTTTGTGGGAATGTGGATGTGCAACTTTAGGAGCTTCTTTCCAATATGCTCAATCTAAACCTAAAATCGAAGAATTAAACGTTCTCTGTAACGCAGCAGAGTTTACTATTAATAAACCTAAAGGGTATGTAGGTAAGGAGTTTCCTCTTGATCTTACAGCAGGAACAGATGCAGCGACGGGCACTAAAGATGCCTCTATTGATTACCATGAGTGGCAAGCAAGTTTATCTCTTTCTTACAGACTCAATATGTTCACTCCCTACATTGGAGTTAAATGGTCTCGTGCAAGCTTTGATTCTGATACAATTCGTATAGCCCAGCCGAGGTTGGTAACACCTGTTGTAGATATTACAACCCTTAACCCAACTATTGCAGGATGCGGCAGTGTAGCTGGAGCTAACACGGAAGGACAGATATCTGATACAATGCAAATCGTCTCCTTGCAATTGAACAAGATGAAATCTAGAAAATCTTGCGGTATTGCAGTAGGAACAACTATTGTGGATGCAGACAAATACGCAGTTACAGTTGAGACTCGCTTGATCGATGAGAGAGCTGCTCACGTAAATGCACAATTCCGCTTCTAACCTCTTCCTGTCACCGACACTTC |

*The complete sequence of the cryptic plasmid (NCBI reference sequence: NC_001372.1) (7502nt) is marked in black; The sequence of the head of the linear cryptic plasmid DNA (400nt) is marked in blue; The full-length sequence (1188nt) of the MOMP gene (GenBank: X52080.1) is marked in orange; The targeting regions (23nt) of the sgRNA are marked in green.

**TABLE S2** Ct values of genes CT and GAPDH, measured by TaqMan Real-Time PCR.

| Sample*  （CT/GAPDH） | CT gene | | | | GAPDH gene | | | | △Ct（CT-GAPDH） |
| --- | --- | --- | --- | --- | --- | --- | --- | --- | --- |
|  | 1 | 2 | 3 | Average | 1 | 2 | 3 | Average |  |
| 1/4 | 23.219 | 24.159 | 24.053 | 23.810 | 21.297 | 21.449 | 21.296 | 21.347 | 2.463 |
| 1/2 | 23.220 | 23.258 | 23.055 | 23.178 | 21.428 | 21.669 | 21.367 | 21.488 | 1.690 |
| 2/2 | 21.312 | 21.901 | 22.182 | 21.798 | 21.102 | 21.358 | 21.535 | 21.332 | 0.466 |
| 3/2 | 22.405 | 22.219 | 21.632 | 22.085 | 22.040 | 22.063 | 21.762 | 21.955 | 0.130 |
| 4/2 | 22.158 | 21.570 | 21.360 | 21.696 | 22.022 | 22.180 | 22.137 | 22.113 | -0.417 |
| 5/2 | 21.627 | 21.964 | 21.430 | 21.674 | 22.433 | 22.432 | 22.351 | 22.405 | -0.731 |
| 6/2 | 21.364 | 22.227 | 22.094 | 21.895 | 22.713 | 23.086 | 23.044 | 22.948 | -1.053 |
| 8/2 | 21.530 | 22.052 | 21.566 | 21.716 | 23.014 | 23.297 | 22.819 | 23.043 | -1.327 |
| 10/2 | 21.414 | 21.480 | 21.771 | 21.555 | 23.086 | 23.319 | 23.395 | 23.267 | -1.712 |
| QCs | 24.425 | 24.705 | 24.186 | 24.439 | 23.101 | 23.457 | 23.210 | 23.256 | 1.183 |

*Samples of CT gene and GAPDH gene mixed at different molar ratios.

**TABLE S3** Data for homogeneity verification.

| Sample* | Cycle Threshold (Ct) values | | | | |
| --- | --- | --- | --- | --- | --- |
|  | 1 | 2 | 3 | Average | Variance |
| 1 | 28.97841263 | 28.94477654 | 29.06727409 | 28.99682109 | 0.004005566 |
| 2 | 29.15361404 | 29.47874451 | 28.32315445 | 28.985171 | 0.355126889 |
| 3 | 29.24864769 | 29.38656807 | 29.0495491 | 29.22825495 | 0.028707344 |
| 4 | 29.40443039 | 29.3094101 | 28.5599575 | 29.091266 | 0.213973749 |
| 5 | 29.11582756 | 28.94363976 | 28.62613678 | 28.89520137 | 0.061708973 |
| 6 | 29.85495186 | 28.6673069 | 27.93613434 | 28.81946437 | 0.93782909 |
| 7 | 28.93575096 | 28.76478958 | 28.96508789 | 28.88854281 | 0.01170131 |
| 8 | 28.92412949 | 28.87216568 | 28.18810654 | 28.66146723 | 0.168727822 |
| 9 | 28.81185722 | 27.64725304 | 28.76254654 | 28.40721893 | 0.433769009 |
| 10 | 28.86351967 | 28.58162498 | 28.61066818 | 28.68527095 | 0.024040332 |

One-way analysis of variance (ANOVA)

| Source of variation | SS | df | MS | F | P-value | F crit |
| --- | --- | --- | --- | --- | --- | --- |
| Between-group variation | 1.505376912 | 9 | 0.167264101 | 0.74685141 | 0.663684578 | 2.392814108 |
| Within-group variation | 4.479180171 | 20 | 0.223959009 |  |  |  |
| Total variation | 5.984557083 | 29 |  |  |  |  |
| F_0.05_ (9，21) =0.746851＜F, there are no significant differences between or within groups, and the QCs have good homogeneity. | | | | | | |

SS: sum of squares, df: degrees of freedom, MS: mean squares.

**TABLE S4** Primers for sequencing the 8630 bp CT sequence.

| Name | Sequences（5’→3’） | Length |
| --- | --- | --- |
| CT-F3 | ATTCCTCTAGTACAAACACCCAC | 23 bp |
| 682F | GCGACTATTCCTTGAGTCATCC | 22 bp |
| 1358F | CCTATAACTGTAGACTCGGCTTG | 23 bp |
| 2042F | CGAGTAGAAGAAGCTGGAG | 19 bp |
| 2699F | TCCAAAGATTACTGACCGCAAC | 22 bp |
| 3360F | TCGGTACATCTGTACAAGGAAC | 22 bp |
| 4037F | CAATGCAACGGGTTATTCAC | 20 bp |
| 4706F | GGATTATTCCCAGACTCTCCTG | 22 bp |
| 5359F | ACCTGAAGAAGAACACATTC | 20 bp |
| 5941F | GATCACTTCTCCTCAACAAC | 20 bp |
| 7154F | ATCCAATCAGATTTCCTTTCGC | 22 bp |
| 7495R | TCCCCAGAACAATAAGAACAC | 21 bp |
| CT-R3 | CAACTGTAACTGCGTATTTGTCT | 23 bp |

**FIGURES**

**
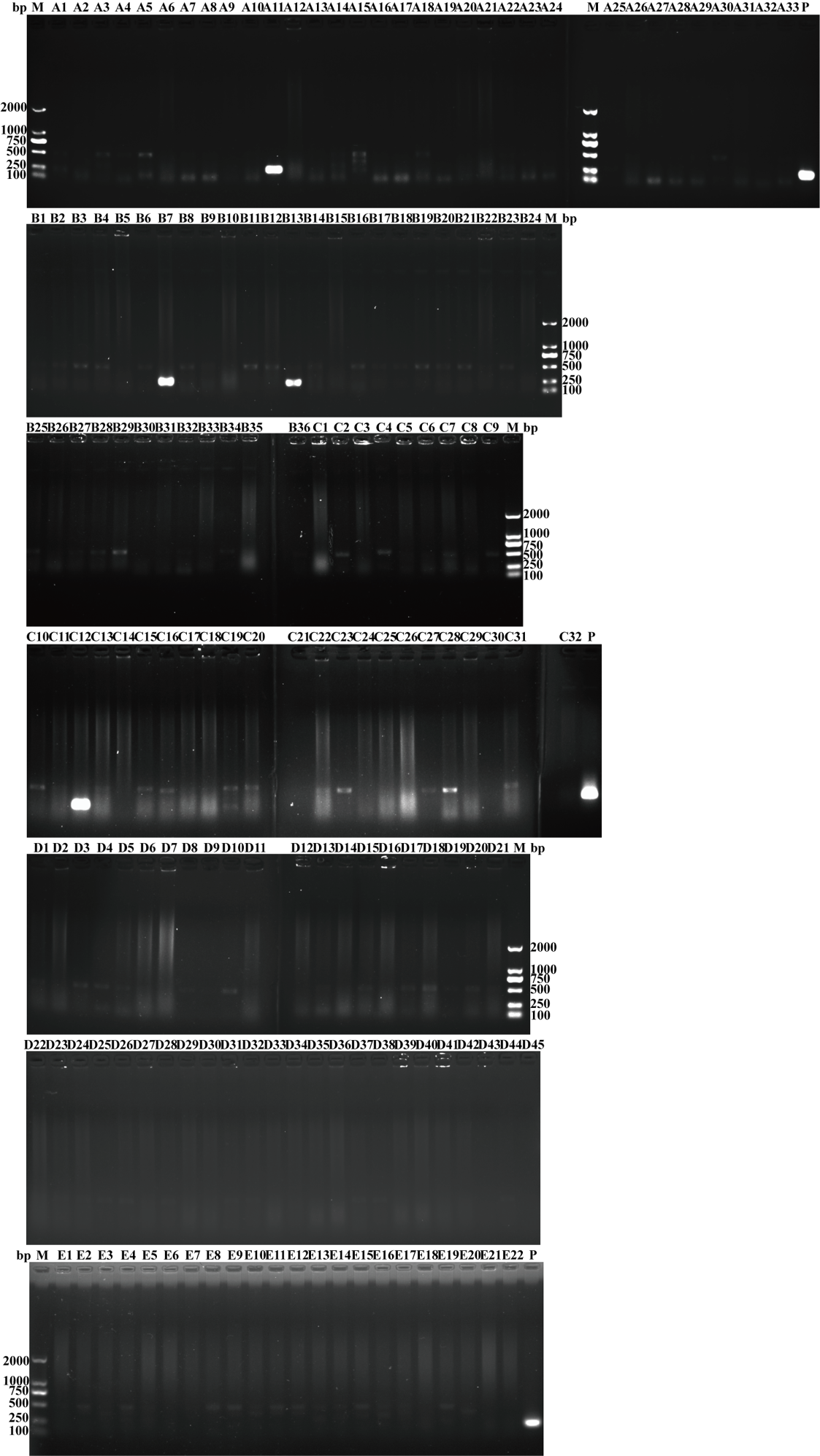
**

**FIG S1** PCR-based agarose gel electrophoresis screening of positive clonal cells. gDNA was extracted from flow-screened monoclonal cells for PCR. Primer pair CT-F1/R1 amplified the head of the insertion sequence (245 bp). The amplification products were analyzed by electrophoresis on 1% agarose gel. The size of the amplicon was as expected, indicating that the corresponding site of the target insertion sequence was successfully integrated into the genome. Lane M: 2000 bp DNA marker; Lane N: Negative control (wild-type HEK293T cells); Lane P: Positive control (The residual mixed pool cells after flow cytometric sorting)

**
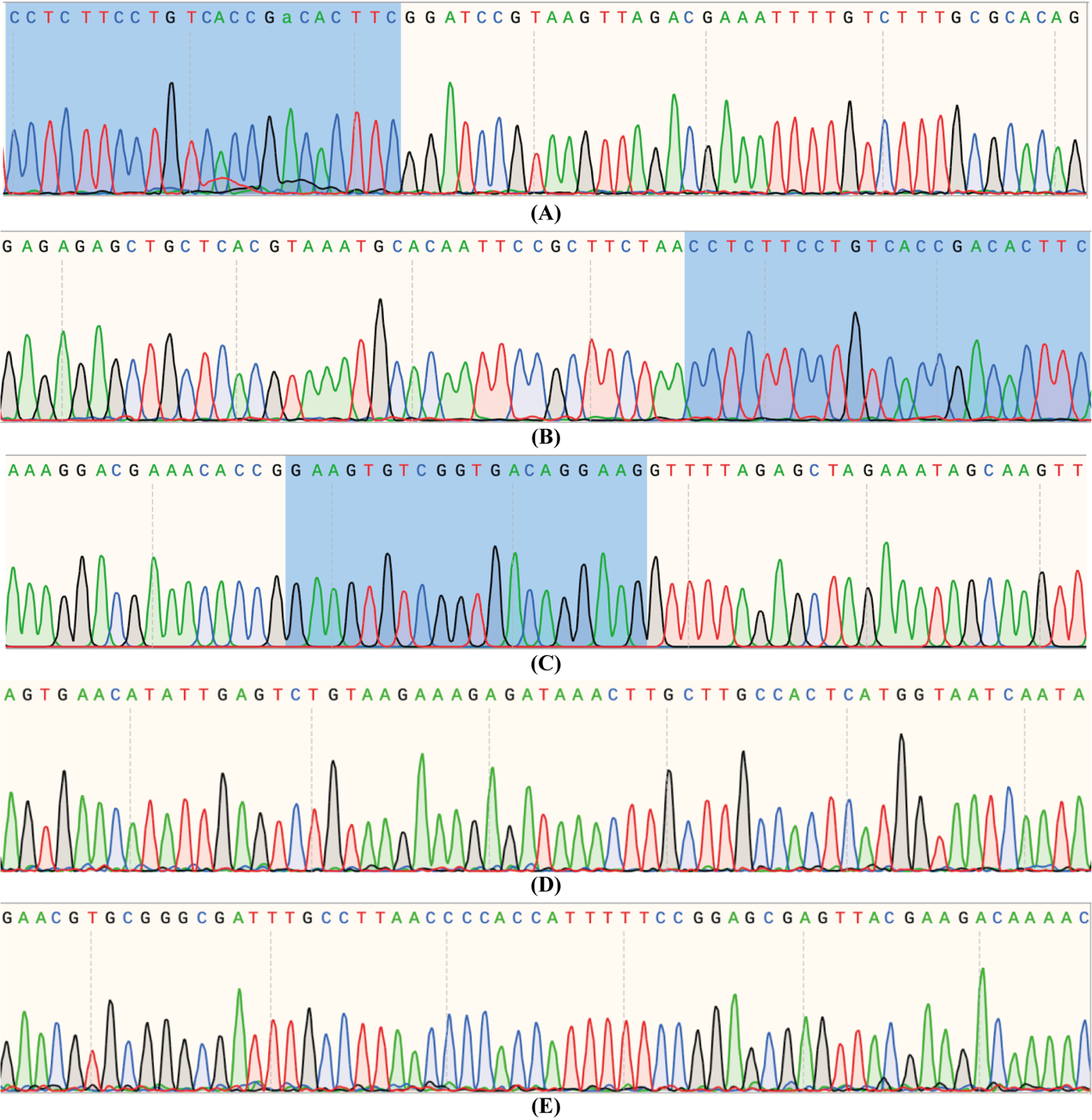
**

**FIG S2** Partial Sanger sequencing results of the recombinant plasmids and the integrated CT sequence of the cell line. (A) Head of the CT DNA insertion sequence in the CT plasmid. (B) Tail of the CT DNA insertion sequence in the CT plasmid. (C) sgRNA sequence of Cas9/sgRNA plasmid. (D) CT DNA insertion sequence-MOMP gene fragment. (E) CT DNA insertion sequence-cryptic plasmid fragment. The blue shaded portion is the sgRNA recognition sequence and sgRNA sequence.

**
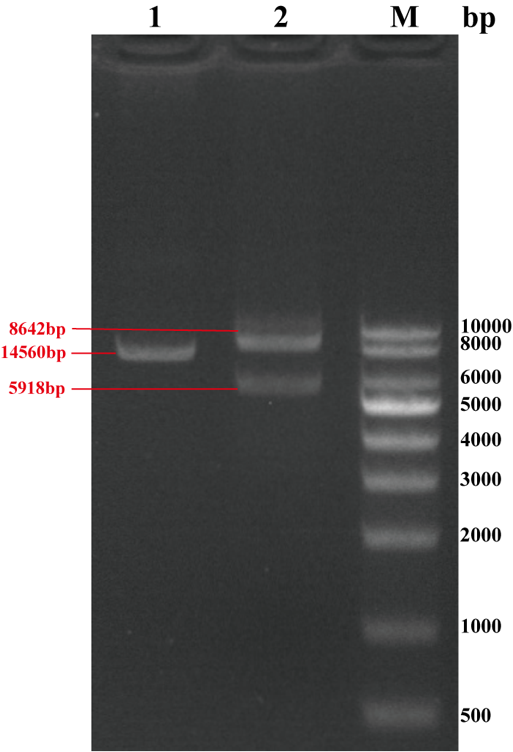
**

**FIG S3** Agarose gel map of double enzyme digestion of CT plasmid. About 500 ng of plasmid was digested at 37°C for 40 minutes and analyzed on 1% agarose gel. Lane 1: CT plasmid; Lane 2: CT plasmid digested by PstI; Lane M: 10000 bp DNA marker.
